# Endosialin and Associated Protein Expression in Soft Tissue Sarcomas: A Potential Target for Anti-Endosialin Therapeutic Strategies

**DOI:** 10.1155/2016/5213628

**Published:** 2016-01-27

**Authors:** Daniel J. O'Shannessy, Hongyue Dai, Melissa Mitchell, Shane Huntsman, Stephen Brantley, David Fenstermacher, Damon R. Reed

**Affiliations:** ^1^Translational Medicine and Diagnostics, Morphotek, Inc., Exton, PA 19341, USA; ^2^M2Gen, Tampa, FL 33612, USA; ^3^H. Lee Moffitt Cancer Center and Research Institute, Department of Biostatistics and Biostatistics, Tampa, FL 33612, USA; ^4^Department of Bioinformatics, Virginia Commonwealth University, Richmond, VA 23284, USA; ^5^Sarcoma Department, H. Lee Moffitt Cancer Center and Research Institute, Tampa, FL 33612, USA; ^6^Chemical Biology and Molecular Medicine Program, H. Lee Moffitt Cancer Center and Research Institute, Tampa, FL 33612, USA; ^7^Adolescent and Young Adult Program, H. Lee Moffitt Cancer Center and Research Institute, Tampa, FL 33612, USA

## Abstract

Endosialin (CD248, TEM-1) is expressed in pericytes, tumor vasculature, tumor fibroblasts, and some tumor cells, including sarcomas, with limited normal tissue expression, and appears to play a key role in tumor-stromal interactions, including angiogenesis. Monoclonal antibodies targeting endosialin have entered clinical trials, including soft tissue sarcomas. We evaluated a cohort of 94 soft tissue sarcoma samples to assess the correlation between gene expression and protein expression by immunohistochemistry for endosialin and PDGFR-*β*, a reported interacting protein, across available diagnoses. Correlations between the expression of endosialin and 13 other genes of interest were also examined. Within cohorts of soft tissue diagnoses assembled by tissue type (liposarcoma, leiomyosarcoma, undifferentiated sarcoma, and other), endosialin expression was significantly correlated with a better outcome. Endosialin expression was highest in liposarcomas and lowest in leiomyosarcomas. A robust correlation between protein and gene expression data for both endosialin and PDGFR-*β* was observed. Endosialin expression positively correlated with PDGFR-*β* and heparin sulphate proteoglycan 2 and negatively correlated with carbonic anhydrase IX. Endosialin likely interacts with a network of extracellular and hypoxia activated proteins in sarcomas and other tumor types. Since expression does vary across histologic groups, endosialin may represent a selective target in soft tissue sarcomas.

## 1. Introduction

Endosialin, also known as Tumor Endothelial Marker-1 (TEM-1) or CD248, was first described as a vascular endothelial cell surface antigen in human fetal fibroblasts [1]. These authors demonstrated monoclonal antibody FB5 reactivity in a significant proportion of tumor blood vessels, including those of sarcoma, while ostensibly being absent from normal tissues. Endosialin is classified as a C-type lectin-like protein and shares both sequence and structural homology with thrombomodulin (CD141) and complement receptor C1qRp (CD93) [2]. All three proteins are involved in, but not limited to, the process of angiogenesis.

Although the domain structure of endosialin is well characterized, the underlying biochemistry of this protein is not well understood. Studies have identified pericytes and reactive fibroblasts, two essential cell types required for tumor survival and growth, as the major sites of expression of endosialin. Pericytes and stromal fibroblasts derive from mesenchymal stem cells [3] and, as such, endosialin has been described as a mesenchymal marker. Pericytes are essential to the process of angiogenesis, especially neoangiogenesis, and serve as scaffolding for endothelial cells but also communicate with endothelial cells by direct physical contact and paracrine signaling pathways [4]. It is now accepted that the formation of solid tumors requires the proliferation of stromal cells to support cancer cell growth, invasion, and metastasis [5] and that the stromal cell compartment comprises a heterogeneous mix of cell types responsible for the formation of blood vessels as well as supporting a microenvironment commensurate with tumor growth and proliferation. The potential for pericytes to contribute to the formation of tissues in addition to vessels has also been described for osteoblasts, chondroblasts, fibroblasts, adipocytes [6, 7], myogenic cells [8], and odontoblasts [9, 10]. The coordinated growth and cross-talk between stromal cell components are critical for establishing a microenvironment that can support the growth and maintenance of tumor cells. This is mediated, at least in part, through direct cell-cell interaction as well as through secreted molecules, including extracellular matrix components (ECM) [11, 12]. Taken together, these data suggest that endosialin plays a role in forming and/or stabilizing the tumor ECM and thus the tumor microenvironment, in addition to its roles in neovascularization and cross-talk between various cell types. Indeed, cell culture studies have demonstrated endosialin to be directly involved in regulating cellular proliferation [13] and in a subset of cells this proliferation appears to involve the PDGFR-*β* pathway [14, 15].

It is now well established that endosialin expression in tumors is not restricted to tumor vessels but is also present in tumor stroma and in some instances expressed by tumor cells themselves. Studies in normal and neoplastic tissue have indicated expression of endosialin in tumor neovasculature within human colorectal cancer [16], breast cancer [17, 18], histiocytomas [19], highly invasive glioblastoma, anaplastic astrocytomas, metastatic carcinomas, and melanoma [20, 21].

Based on the important role of stroma in supporting tumor growth and the activity of endosialin in supporting tumor stromal cell functions, clinical studies using a humanized monoclonal antibody, ontuxizumab (MORAb-004), are currently underway to determine the safety and clinical activity of blocking endosialin in patients with various cancer types [22]. Sarcomas are a diverse collection of cancers of mesenchymal origin which can affect all ages. Importantly, endosialin expression has been characterized directly on sarcoma tumor cells [23, 24]. While overall incidence of sarcomas is 1% of all newly diagnosed cancers, it is relatively more common in younger ages consisting of 15% of childhood cancers and 8% of cancers in young adults. Metastatic sarcomas have a poor prognosis with few advances in recent decades [25, 26]. Phase II studies have demonstrated few active agents even with a relatively low efficacy bar of 40% progression-free survival at 4 months [27]. A recently accrued but not yet reported phase II, randomized clinical trial (NCT01574716) investigated the addition of ontuxizumab to gemcitabine and docetaxel in adult sarcoma patients stratified by diagnosis into one of four groups of leiomyosarcoma, liposarcoma, undifferentiated, and other soft tissue sarcomas.

We therefore undertook a preclinical assessment into the relative expression of endosialin and PDGFR-*β* in a novel, retrospective cohort of soft tissue sarcoma patients by immunohistochemistry (IHC) and gene expression. We further compared expression to other potential endosialin-associated proteins and with clinical characteristics including outcomes to address efficacy as well as enhance the development of these biomarkers in sarcoma and other patient populations.

## 2. Materials and Methods

### 2.1. The Total Cancer Care Protocol

In 2006, Moffitt Cancer Center launched Total Cancer Care (TCC), an observational research study designed with the objective of following patients throughout their lifetime to identify and meet patients' needs [28], consenting to the TCC protocol that allows for lifetime follow-up, data sharing, use of tumor specimens for research and biomarker analyses, and the ability to recontact patients if there is a finding that could influence patient care, including clinical trials. The TCC protocol is a network of 17 hospitals, all of which adhere to the same procedures for collection and shipment of tissue for molecular analysis and abstraction of longitudinal clinical data. As of May 2015, over 123,000 patients have consented to the TCC protocol with more than 39,000 fresh frozen tumor specimens collected and molecular data on nearly half of the specimens. To ensure compliance with HIPAA and Human Subject Research requirements, an oversight committee monitors TCC operations and procedures.

### 2.2. Identification of Sarcoma Samples

Expression of endosialin and PDGFR-*β* was calculated as described for all tumor samples that had a corresponding Affymetrix microarray. Sarcoma samples were identified and subtyped based on the pathology/histology data available in the Total Cancer Care Data Warehouse. Sarcoma tumor samples were ordered by log_2_ expression values for both endosialin and PDGFR-*β* to determine quartile cutoffs for both genes. Within each quartile, twenty samples were selected in an effort to understand the levels of gene expression that correlated with positive IHC *H*-scores. This selection of samples in each quartile resulted in the identification of FFPE samples with log_2_ expression values ranging from 12.2 to 5.6 for PDGFR-*β* and from 12.9 to 5.7 for endosialin. In total, 94 independent FFPE samples were selected for IHC analysis for both endosialin and PDGFR-*β*.

### 2.3. Clinical Data

Samples were identified through Health Information Resources and consisted of deidentified samples. Clinical data including diagnosis and stage were abstracted electronically from the Data Warehouse. To confirm accurate information, deidentified pathology reports with all PHI redacted were reviewed. All samples were categorized as undifferentiated, leiomyosarcoma, liposarcoma, and other.

### 2.4. Immunohistochemistry—Endosialin and PDGFR-*β*

Immunohistochemistry (IHC) for endosialin was performed as previously described [29] using monoclonal antibody 9G5 on 5 *μ*m FFPE specimens. For the detection of PDGFR-*β*, monoclonal antibody clone 28E1 (1 : 50 dilution; Cell Signaling) was incubated for 30 min as primary or Flex Rabbit Negative (Dako) as the negative control. Primary antibody was detected using Envision Flex HRP (Dako) for 30 min followed by Flex DAB+ Chromogen (Dako). All tissue sections were counterstained with hematoxylin for 2 min. The H&E and IHC stained slides were reviewed by a single ABP-certified pathologist using a protocol derived from standard methods for *H*-scoring [30–34]. The entire slide was scanned at 10x magnification, and the percentage of each degree of staining intensity in the tumor cells was determined: *H*-score = (%0+)+(%1+)+(%2+)+(%3+). Each slide was examined twice at separate sessions to ensure that intraobserver variation was not a factor. Slides were evaluated without access to patient demographics or clinical information, and the original histopathologic diagnoses were not reviewed until the analysis was complete. No discrepancies with the original diagnoses were identified.

### 2.5. Gene Expression Data

Fresh frozen tumor samples from TCC consented subjects were collected and RNA extracted. Sample amplification, labeling, and microarray processing were performed by the Rosetta Inpharmatics Gene Expression Laboratory (Seattle, WA). Samples were amplified and labeled using the NuGEN Ovation WB protocol and were hybridized to the Rosetta/Merck Custom Affymetrix microarray, the HuRSTA-2a520709 Affymetrix array (GEO GPL15048), following the standard Affymetrix protocol. Expression arrays were normalized using the IRON method (Iterative Rank-Order Normalization) [35]. The IRON array processing pipeline employs RMA background subtraction, Tukey's Biweight probeset summarization, and a novel pairwise (sample versus reference) IRON method that is able to largely handle violations of the symmetry assumption implicit in quantile normalization and traditional pairwise normalization algorithms. Endosialin and PDGFR-*β* gene expressions were measured by probes* merck-NM_020404_at* and* merck-NM_002609_at,* respectively, and were log_2_ transformed. For other genes assessed in this work (FN1, COL1A1, COL1A2, COL4A1, COL4A2, COL4A3, COL4A4, COL4A5, COL4A6, CA9, EPAS1, and HSPG2), the corresponding probes are provided in Table 1. If a gene had multiple probes on the array, the log_2_ expression levels were averaged.

### 2.6. Statistical Methods

The log_2_ gene expression levels and IHC discrete values (0, 1, 2, and 3) were used to compute the correlation (Pearson) coefficients between the mRNA and protein levels across sarcoma patients. Spearman correlation yielded very similar results. The R stats package (http://cran.r-project.org/web/packages/) was used to test expression and IHC levels versus sarcoma histotypes. More specifically, we used linear model and ANOVA analysis from the R packages. The survival package from R was used to generate Kaplan-Meier curves and perform log-rank test and the Cox proportional hazards modeling.

## 3. Results

**Figure 1:**
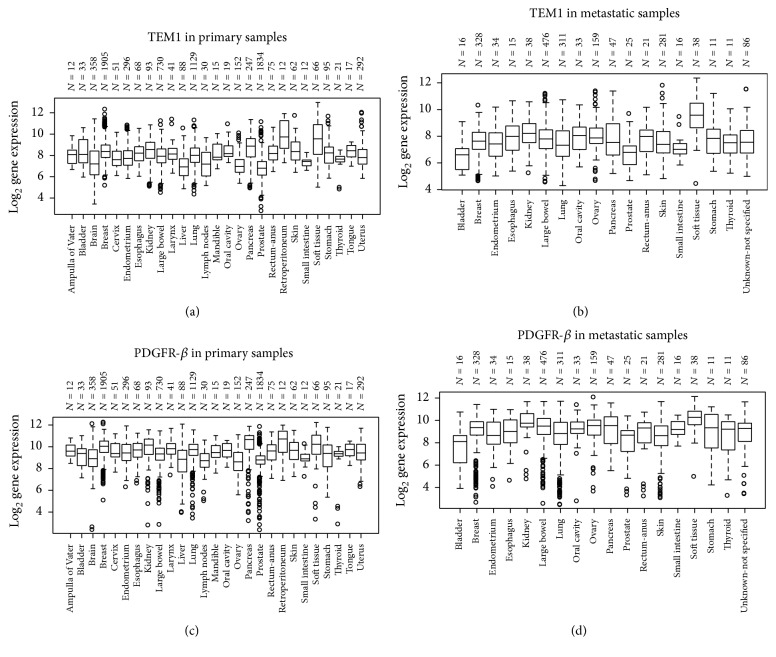
Expression of endosialin and PDGFR-*β* across numerous solid tumors. Gene expression of endosialin and PDGFR-*β* was evaluated across all tumor types in the Total Cancer Care® (TCC) Data Warehouse in a total of 15,820 samples. The distribution of the expression level in each tumor type is represented by box plots, with the middle horizontal line representing the median and the box representing the 25th to 75th percentiles. (a) Endosialin in primary tumors; (b) endosialin in metastatic tumors; (c) PDGFR-*β* in primary tumors; and (d) PDGFR-*β* in metastatic tumors. Both endosialin and PDGFR-*β* are highly expressed in soft tissue tumors which are mostly comprised of sarcomas.

**Figure 2:**
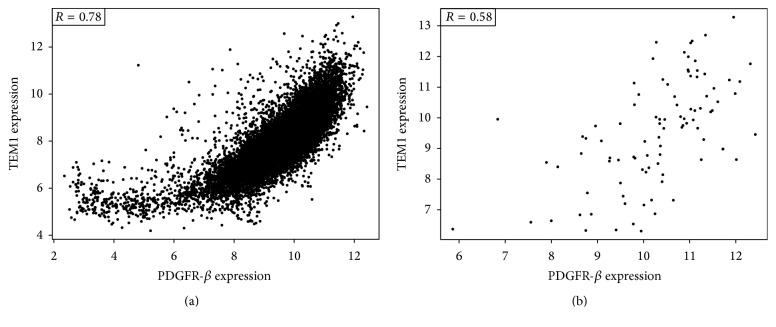
Correlation between endosialin and PDGFR-*β* gene expression. Endosialin (TEM-1) and PDGFR-*β* expression levels were assessed across all 15,820 samples in the TCC Data Warehouse (a) and in the subset of 94 sarcoma samples (b) and were shown to be highly correlated: *R* = 0.78 and 0.58, respectively.

We initially evaluated the expression of endosialin and PDGFR-*β* in both primary tumor samples and metastatic lesions across a variety of tumor types in a total of 15,820 samples from the TCC cohort (Figure 1). As can be seen from these data, the level of expression of these two genes varies across tumor types but both genes are ubiquitously expressed (Figures 1(a) and 1(c)). These data also demonstrate that expression of both genes is retained in metastatic lesions (Figures 1(b) and 1(d)) at levels comparable to the primary tumor. There are consistently high levels of expression of both endosialin and PDGFR-*β* in soft tissue tumors, which were mostly sarcomas, relative to the other tumor types assessed. Across the entire data set there was a high correlation between the expression of endosialin and PDGFR-*β* (Figure 2(a)).

To more thoroughly evaluate sarcomas, samples from 94 patients for which all data was available were identified (Table 2) and used in the present study. The mean age of the patients was 59 with a range of 19–90 years. Samples were categorized by histology in accordance with, but distinct from, the active randomized, blinded clinical trial (NCT01574716) with gemcitabine and docetaxel with or without MORAb-004 into 4 subgroups: leiomyosarcoma (28 subjects), liposarcoma (22 subjects), undifferentiated (34 subjects), and other (10 subjects: 4 with angiosarcoma and 6 with synovial sarcoma). The majority of subjects in this study presented with high grade tumors and were classified as stage III at diagnosis. The majority of samples (55; 59%) were primary tumor resections, with the remainder representing relapsed or progressive metastatic samples (39; 41%). As shown in Figure 2(b), the expression of endosialin and PDGFR-*β* is highly correlated across these 94 sarcoma samples.

**Figure 3:**
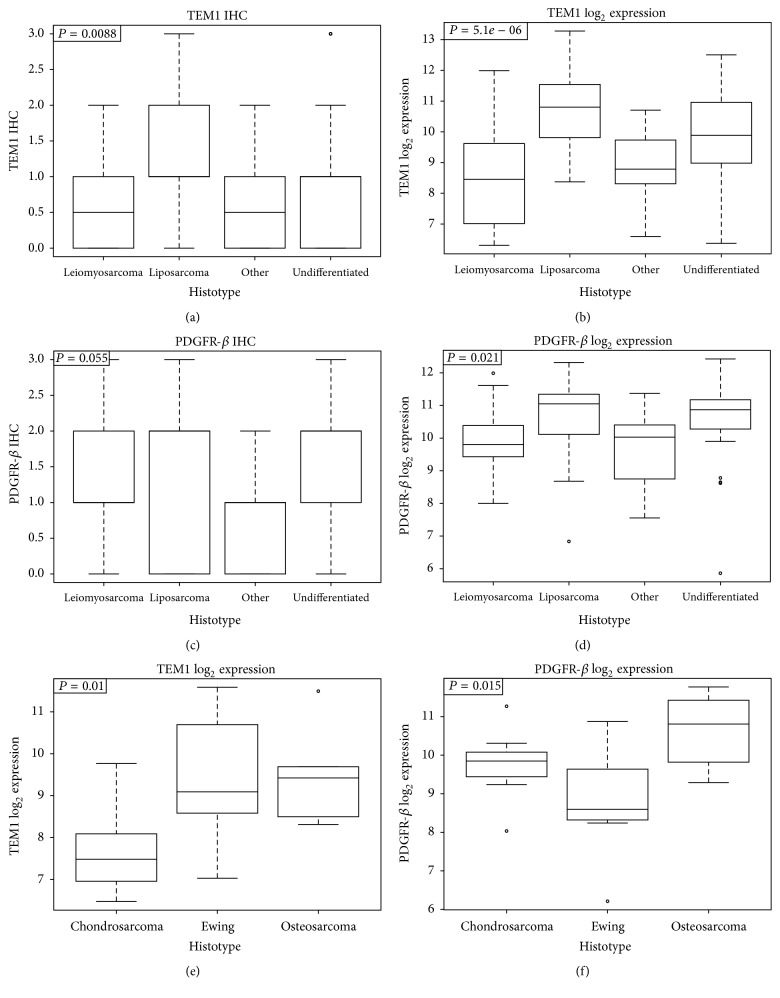
Expression of endosialin and PDGFR-*β* across sarcoma histotypes. Gene expression and semiquantitative IHC data for endosialin and PDGFR-*β* in different histotypes of sarcoma. The distribution of the expression level in each subtype is represented by box plots, with the middle horizontal line representing the median and box representing 25th to 75th percentiles. (a) Endosialin IHC; (b) endosialin gene expression; (c) PDGFR-*β* IHC; (d) PDGFR-*β* gene expression; (e) and (f) endosialin and PDGFR-*β* gene expression in bone sarcoma subtypes. *P* values represent multigroup ANOVA *P* values.

A good correlation was observed across the entire sarcoma sample set for endosialin and PDGFR-*β* expression determined by both IHC and gene expression analysis (Figures 3(a)–3(d)). The relative expression across all 4 sarcoma subgroups for both endosialin and PDGFR-*β*, both by IHC and gene expression analysis, shows that liposarcoma had slightly higher IHC scores and gene expression levels than the other subgroups analyzed (see Table 3 for pairwise comparisons). Bone sarcomas demonstrated consistently high endosialin and PDGFR-*β* expression with chondrosarcoma having lower expression (Figures 3(e)–3(f)).

**Figure 4:**
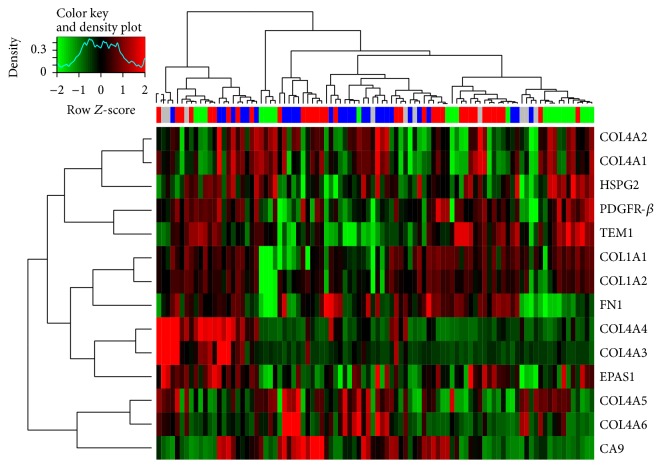
Heat map of expression of potential endosialin interacting proteins.

Since a number of potential interacting proteins and pathways have been described in the literature to be involved in endosialin biology, we further examined the expression of a number of extracellular matrix and hypoxia related genes.  Figure 4 shows the heat map derived from these analyses with no obvious clustering of samples or histotypes. However, a number of positive correlations (*P* < 0.02) were identified between endosialin or PDGFR-*β* IHC expression (Table 4) versus gene expression of this gene set, and by PDGFR-*β* gene expression versus gene expression of the gene set (Table 5). Endosialin was most highly positively correlated to PDGFR-*β* and HSPG2 (heparin sulphate proteoglycan 2) and negatively correlated to CAIX (carbonic anhydrase IX). PDGFR-*β* was most highly positively correlated to FN1 (fibronectin) and negatively correlated to COL4A5 (collagen, type IV, alpha 5).

**Figure 5:**
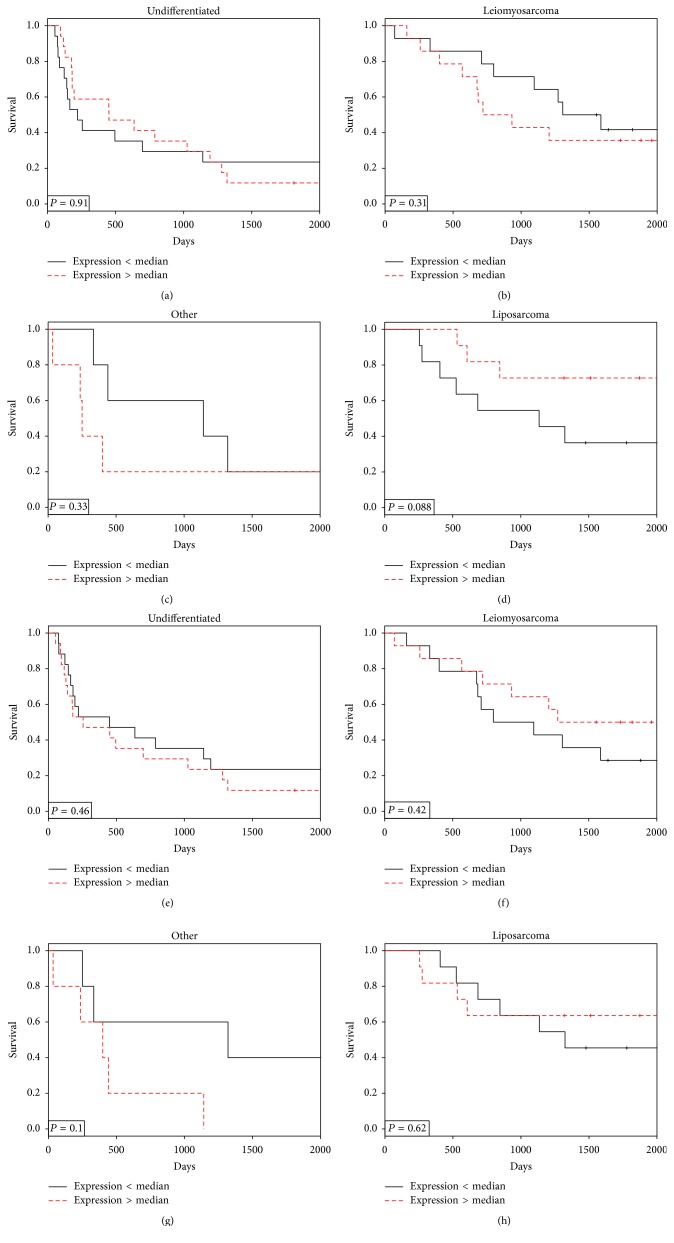
Endosialin and PDGFR-*β* gene expression and outcomes in sarcoma histotypes. Endosialin ((a)–(d)) and PDGFR-*β* ((e)–(h)) expression* versus* overall survival in different histological subtypes of sarcoma. (a) and (e) Undifferentiated (*N* = 34); (b) and (f) leiomyosarcoma (*N* = 28); (c) and (g) other (angiosarcoma and synovial sarcoma) (*N* = 10); and (d) and (h) liposarcoma (*N* = 22).

**Figure 6:**
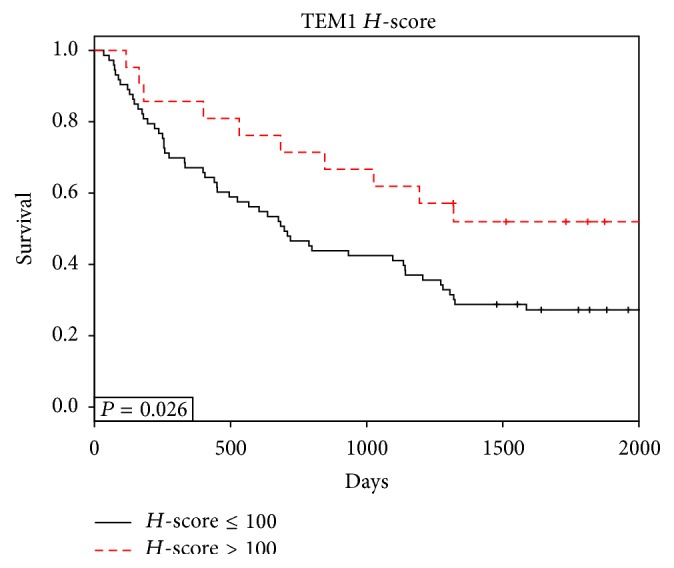
Endosialin protein expression (IHC) and outcomes in sarcoma. IHC was performed as described and slides scored by a Board-certified pathologist. The figure represents endosialin *H*-score versus overall survival in the 94 sarcoma patients cohort. High *H*-score correlates to improved outcome.

Finally, the relationship between endosialin gene expression and survival was evaluated relative to sarcoma histotype (Figure 5). While not significant (*P* = 0.09) there was a trend towards a survival benefit for higher endosialin gene expression in the liposarcoma subtype. It should be noted that these analyses were performed by bifurcation of the histotypes at the median of endosialin expression. In contrast, endosialin protein expression, determined by semiquantitative IHC using the *H*-score, was significantly (*P* = 0.026) associated with survival using an *H*-score cut point at the 75th percentile (*H*-score = 100); that is, increased endosialin expression resulted in better survival for the entire cohort (Figure 6). Further, by Cox proportional hazards analysis which does not depend on any threshold, the log⁡ (hazards  ratio) was −0.0039 ± 0.0016 (*P* = 0.015). PDGFR-*β* *H*-score, on the other hand, was not significant by the Cox model (*P* = 0.44).

## 4. Discussion

To our knowledge, this 15,820-sample cohort across 27 different cancer location types represents the largest reported for endosialin and PDGFR-*β* expression. Soft tissue tumors, primarily composed of sarcomas, are at the high end of expression of endosialin which might support evaluation of this tumor type in a clinical setting. Additionally, expression of both endosialin and PDGFR-*β* was maintained in metastatic lesions providing a potential target for advanced disease. This expression may be a reflection of the integral roles endosialin and PDGFR-*β* have in tumor biology. In metastatic lesions, both endosialin and PDGFR-*β* were expressed more highly in soft tissue tumors than in any of the other tumor types assessed. It is also important to note that there was a very high correlation between the expression of these two genes across the entire 15,820-sample set, as well as the 94-sarcoma cohort.

It is now generally recognized that endosialin expression is present in tumor vasculature, specifically pericytes, as well as in activated macrophages within the tumor stroma [36]. The expression of endosialin in sarcomas is well documented in cell lines, including stem cell enriched side populations [37], and expression was maintained when cell lines were implanted* in vivo *and metastasized [24]. Further, endosialin expression, determined by IHC, in archived sarcoma samples has been reported [23]. In the present study, we demonstrated excellent concordance of endosialin expression by IHC and by gene expression analysis in a sarcoma cohort and both agree with and extend previous studies.

Agents targeting endosialin are now in the clinic with safety data reported from a phase I trial of the anti-endosialin therapeutic monoclonal antibody ontuxizumab (MORAb-004) in advanced solid tumors [22]. Overall the agent was well tolerated with fatigue (47.2%), headache (36.1%), pyrexia (22.2%), chills (19.4%), and nausea (dose limiting toxicity, 13.9%) being reported and with infusion related reactions manageable with premedications. This agent is also being investigated in a recently enrolled phase II sarcoma trial.

Having access to the expression data for this sarcoma cohort allowed us to investigate potential gene associations beyond PDGFR-*β* for a number of literature-reported endosialin binding partners and associated pathways. For example, studies demonstrated that fibronectin and collagens types I and IV are binding partners of endosialin [38]. Evidence for the criticality of endosialin expression in tumor growth and progression comes from the findings in glioblastoma cells and placental fibroblasts that endosialin expression is upregulated by hypoxia inducible factor-2 (HIF-2) [39], produced as a result of rapid proliferation of tumor cells generating areas of hypoxia leading to the expression of HIF-1 and HIF-2, upregulated gene expression, and vascular remodeling. Animal models have further demonstrated that cancer invasion is stimulated by stromal microenvironments similar to those present in wound healing [40]. This observation suggests that growth factors implicated in wound healing such as transforming growth factor-*β* (TGF-*β*) and PDGFR-*β* may also play a role in altering the stromal host compartment in support of cancer.

Of the genes examined, the most significant correlations with endosialin expression were seen with PDGFR-*β*, as expected, and HSPG2 and a negative correlation with CAIX. A significant but weaker negative correlation was also observed for COL4A6. Interestingly perhaps is that no correlation was seen between endosialin expression and HIF-2 expression. As noted above, HIF-2 expression has been reported to upregulate endosialin expression and also regulates CAIX expression. Further studies are clearly needed to elucidate the regulation of expression of endosialin. The expression of PDGFR-*β* was highly correlated to fibronectin and highly negatively correlated to COL4A5. It is worth noting that endosialin expression was also negatively correlated to COL4A5 but did not reach significance. Similar to the results with endosialin, PDGFR-*β* was significantly negatively correlated to COL4A6 as well as COL1A1 and COL1A2. While inconclusive, these data combined are suggestive of an interaction, either direct or indirect, between endosialin and PDGFR-*β* and with extracellular matrix components (ECM) such as fibronectin and collagens. Indeed, in a recent study [41] on archival colorectal cancer specimens using fluorescent immunohistochemistry approaches, the architectural expression (tumor, stroma, and vessel) of endosialin, ECM components (fibronectin, collagens types I and IV), PDGFR-*β*, HIF-2, and CAIX demonstrated a complex interplay between endosialin and the tumor microenvironment. Importantly, this prior work also demonstrated that the architectural expression and quantitation of these endosialin-associated proteins was prognostic in colorectal cancer.

In the present sarcoma cohort, based on gene expression data, we were unable to demonstrate a clear correlation with endosialin-associated expression and overall survival. However, unlike the study on colorectal cancer, and absent laser microdissection, gene expression studies are burdened by averages with no architectural or contextual information. In the liposarcoma subcohort, which showed higher endosialin gene expression relative to the other subcohorts evaluated, there was a distinct trend, though not significant, towards increased survival with increased endosialin expression. Interestingly however, when endosialin expression by IHC was assessed across the entire 94-patient cohort, it was significantly predictive of survival, that is, prognostic, again with higher expression correlating with improved survival.

## 5. Conclusions

Taken together, the present results support a relationship between endosialin and PDGFR-*β* in concert with the ECM. The exact nature of these relationships and their influence on the tumor microenvironment and ultimately tumor growth and metastasis are yet to be elucidated. However, the present data on a relatively large sarcoma cohort supports the evaluation of anti-endosialin therapies, such as ontuxizumab (MORAb-004), in the clinical setting of sarcoma.

## Figures and Tables

**Table 1:** Probe sets used in the present study.

Gene	Probe set(s)
FN1	merck-NM_054034_a_at

COL1A1	merck-NM_000088_at; merck-BC036531_a_at; merck2-DQ893571_at

COL1A2	merck-BC042586_a_at; merck-CF125809_s_at; merck2-NM_000089_at; merck2-AI983991_a_at; merck2-AK226074_a_at; merck2-BY794944_a_at

COL4A1	merck-NM_001845_at; merck-AK058157_at; merck2-AL572880_at; merck2-M10940_at

COL4A2	merck-M24766_a_at; merck2-NM_001846_at; merck2-BM546367_a_at; merck2-BQ014598_a_at

COL4A3	merck-M81379_a_at; merck-NM_000091_at

COL4A4	merck-NM_000092_at; merck-BC065843_at; merck2-D17391_at

COL4A5	merck-NM_033381_at; merck-AV659759_at; merck2-AL049291_at

COL4A6	merck-ENST00000345014_at; merck-NM_001847_at; merck-ENST00000358735_at; merck-AL049300_at

CAIX	merck-NM_001216_at; merck2-DQ892208_at

EPAS1	merck-NM_001430_at; merck-BC070392_at; merck-BC026731_at; merck-AK023572_at

HSPG2	merck-X62515_a_at

**Table 2:** Clinical characteristics of 94 sarcoma patients.

Characteristic		*N*	%
Age (years)	Mean	59	
	SD	16.5	
	Range	19–90	

Histotype	Leiomyosarcoma	28	29.8%
	Liposarcoma	22	23.4%
	Undifferentiated	34	36.2%
	Other	10	10.6%

Stage	I	1	1.1%
	II	9	9.6%
	III	84	89.4%

Site of sample	Primary	55	58.5%
	Metastatic	39	41.5%

Vitals	Alive	28	29.8%
	Dead	66	70.2%

**(a):** 

	Undifferentiated	Leiomyosarcoma	Other	Liposarcoma
	TEM-1 IHC			
Undifferentiated	NA	3.74*E* − 01	2.12*E* − 01	2.49*E* − 02
Leiomyosarcoma	NA	NA	5.79*E* − 01	4.89*E* − 03
Other	NA	NA	NA	6.55*E* − 03
Liposarcoma	NA	NA	NA	NA

	TEM1 log_2_ expression			
Undifferentiated	NA	9.80*E* − 04	8.00*E* − 02	3.65*E* − 02
Leiomyosarcoma	NA	NA	4.30*E* − 01	3.51*E* − 06
Other	NA	NA	NA	9.41*E* − 04
Liposarcoma	NA	NA	NA	NA

**(b):** 

	Undifferentiated	Leiomyosarcoma	Other	Liposarcoma
	PDGFR-*β* IHC			
Undifferentiated	NA	9.08*E* − 02	7.12*E* − 03	2.91*E* − 01
Leiomyosarcoma	NA	NA	1.01*E* − 01	7.16*E* − 01
Other	NA	NA	NA	1.21*E* − 01
Liposarcoma	NA	NA	NA	NA

	PDGFR-*β* log_2_ expression			
Undifferentiated	NA	2.11*E* − 02	4.22*E* − 02	7.84*E* − 01
Leiomyosarcoma	NA	NA	5.01*E* − 01	2.13*E* − 02
Other	NA	NA	NA	4.90*E* − 02
Liposarcoma	NA	NA	NA	NA

**(c):** 

	Osteosarcoma	Ewing	Chondrosarcoma
	TEM1 log_2_ expression		
Osteosarcoma	NA	9.43*E* − 01	6.37*E* − 03
Ewing	NA	NA	1.17*E* − 02
Chondrosarcoma	NA	NA	NA

	PDGFR-*β* log_2_ expression		
Osteosarcoma	NA	1.65*E* − 02	7.00*E* − 02
Ewing	NA	NA	8.01*E* − 02
Chondrosarcoma	NA	NA	NA

**Table:** (a) Endosialin IHC correlation with gene expression

Gene	Correlation	*P* value
PDGFR-*β*	0.27	9.63*E* − 03
TEM1	0.61	8.27*E* − 11
FN1	−0.09	3.82*E* − 01
COL1A1	0.14	1.85*E* − 01
COL1A2	0.06	5.95*E* − 01
COL4A1	0.00	9.73*E* − 01
COL4A2	−0.02	8.83*E* − 01
COL4A3	−0.14	1.68*E* − 01
COL4A4	−0.08	4.41*E* − 01
COL4A5	−0.12	2.60*E* − 01
COL4A6	−0.22	3.18*E* − 02
CAIX	−0.29	4.65*E* − 03
EPAS1	0.08	4.22*E* − 01
HSPG2	0.30	3.19*E* − 03

**Table:** (b) PDGFR-*β* IHC correlation with gene expression

Gene	Correlation	*P* value
PDGFR-*β*	0.43	1.76*E* − 05
TEM1	0.26	1.25*E* − 02
FN1	0.44	1.07*E* − 05
COL1A1	0.32	1.75*E* − 03
COL1A2	0.32	1.59*E* − 03
COL4A1	−0.16	1.31*E* − 01
COL4A2	−0.19	7.32*E* − 02
COL4A3	−0.06	5.38*E* − 01
COL4A4	0.08	4.33*E* − 01
COL4A5	−0.42	2.43*E* − 05
COL4A6	−0.30	3.35*E* − 03
CAIX	−0.22	3.45*E* − 02
EPAS1	0.21	3.91*E* − 02
HSPG2	−0.05	6.37*E* − 01

**(a):** 

*P* values	TEM1	PDGFR-*β*	FN1	COL1A1	COL1A2	COL4A1	COL4A2	COL4A3	COL4A4	COL4A5	COL4A6	CA9	EPAS1	HSPG2
TEM1	NA	1.20*E* − 09	9.38*E* − 01	2.24*E* − 05	1.56*E* − 02	6.38*E* − 01	8.16*E* − 01	6.77*E* − 01	6.02*E* − 01	1.39*E* − 02	1.28*E* − 04	8.09*E* − 04	1.60*E* − 02	4.40*E* − 05
PDGFR-*β*	1.20*E* − 09	NA	1.64*E* − 01	2.10*E* − 08	2.07*E* − 04	4.03*E* − 02	4.53*E* − 02	7.50*E* − 02	2.14*E* − 02	1.49*E* − 02	3.04*E* − 03	2.76*E* − 01	2.44*E* − 05	2.69*E* − 04
FN1	9.38*E* − 01	1.64*E* − 01	NA	7.02*E* − 04	5.23*E* − 06	4.21*E* − 01	1.81*E* − 01	3.19*E* − 01	3.21*E* − 02	2.32*E* − 02	2.82*E* − 01	3.20*E* − 02	6.48*E* − 03	1.09*E* − 01
COL1A1	2.24*E* − 05	2.10*E* − 08	7.02*E* − 04	NA	0.00*E* + 00	3.65*E* − 01	2.05*E* − 01	3.79*E* − 01	2.91*E* − 02	1.21*E* − 01	2.29*E* − 02	2.85*E* − 01	1.03*E* − 03	1.10*E* − 01
COL1A2	1.56*E* − 02	2.07*E* − 04	5.23*E* − 06	0.00*E* + 00	NA	1.73*E* − 01	1.87*E* − 01	2.03*E* − 01	1.51*E* − 02	1.08*E* − 01	3.46*E* − 01	5.73*E* − 01	4.64*E* − 04	5.31*E* − 01
COL4A1	6.38*E* − 01	4.03*E* − 02	4.21*E* − 01	3.65*E* − 01	1.73*E* − 01	NA	0.00*E* + 00	6.51*E* − 01	8.30*E* − 01	1.12*E* − 02	1.55*E* − 01	7.99*E* − 02	5.38*E* − 03	7.71*E* − 06
COL4A2	8.16*E* − 01	4.53*E* − 02	1.81*E* − 01	2.05*E* − 01	1.87*E* − 01	0.00*E* + 00	NA	7.24*E* − 01	8.02*E* − 01	8.30*E* − 04	4.86*E* − 03	5.46*E* − 02	7.42*E* − 03	1.23*E* − 06
COL4A3	6.77*E* − 01	7.50*E* − 02	3.19*E* − 01	3.79*E* − 01	2.03*E* − 01	6.51*E* − 01	7.24*E* − 01	NA	0.00*E* + 00	2.31*E* − 01	7.70*E* − 01	9.63*E* − 01	3.23*E* − 04	7.26*E* − 01
COL4A4	6.02*E* − 01	2.14*E* − 02	3.21*E* − 02	2.91*E* − 02	1.51*E* − 02	8.30*E* − 01	8.02*E* − 01	0.00*E* + 00	NA	4.18*E* − 01	9.87*E* − 01	5.10*E* − 01	1.39*E* − 04	8.37*E* − 01
COL4A5	1.39*E* − 02	1.49*E* − 02	2.32*E* − 02	1.21*E* − 01	1.08*E* − 01	1.12*E* − 02	8.30*E* − 04	2.31*E* − 01	4.18*E* − 01	NA	0.00*E* + 00	1.11*E* − 01	6.47*E* − 01	8.54*E* − 01
COL4A6	1.28*E* − 04	3.04*E* − 03	2.82*E* − 01	2.29*E* − 02	3.46*E* − 01	1.55*E* − 01	4.86*E* − 03	7.70*E* − 01	9.87*E* − 01	0.00*E* + 00	NA	3.28*E* − 02	6.78*E* − 01	1.81*E* − 01
CA9	8.09*E* − 04	2.76*E* − 01	3.20*E* − 02	2.85*E* − 01	5.73*E* − 01	7.99*E* − 02	5.46*E* − 02	9.63*E* − 01	5.10*E* − 01	1.11*E* − 01	3.28*E* − 02	NA	3.81*E* − 01	3.84*E* − 01
EPAS1	1.60*E* − 02	2.44*E* − 05	6.48*E* − 03	1.03*E* − 03	4.64*E* − 04	5.38*E* − 03	7.42*E* − 03	3.23*E* − 04	1.39*E* − 04	6.47*E* − 01	6.78*E* − 01	3.81*E* − 01	NA	4.83*E* − 04
HSPG2	4.40*E* − 05	2.69*E* − 04	1.09*E* − 01	1.10*E* − 01	5.31*E* − 01	7.71*E* − 06	1.23*E* − 06	7.26*E* − 01	8.37*E* − 01	8.54*E* − 01	1.81*E* − 01	3.84*E* − 01	4.83*E* − 04	NA

**(b):** 

Correlations	TEM1	PDGFR-*β*	FN1	COL1A1	COL1A2	COL4A1	COL4A2	COL4A3	COL4A4	COL4A5	COL4A6	CA9	EPAS1	HSPG2
TEM1	1	0.58	−0.01	0.42	0.25	0.05	0.02	−0.04	0.05	−0.25	−0.38	−0.34	0.25	0.41
PDGFR-*β*	0.58	1	0.14	0.54	0.37	0.21	0.21	0.18	0.24	−0.25	−0.3	−0.11	0.42	0.37
FN1	−0.01	0.14	1	0.34	0.45	−0.08	−0.14	0.1	0.22	−0.23	−0.11	0.22	0.28	−0.17
COL1A1	0.42	0.54	0.34	1	0.8	−0.09	−0.13	0.09	0.23	−0.16	−0.23	−0.11	0.33	0.17
COL1A2	0.25	0.37	0.45	0.8	1	−0.14	−0.14	0.13	0.25	−0.17	−0.1	0.06	0.35	0.07
COL4A1	0.05	0.21	−0.08	−0.09	−0.14	1	0.91	0.05	0.02	0.26	0.15	0.18	0.28	0.44
COL4A2	0.02	0.21	−0.14	−0.13	−0.14	0.91	1	0.04	0.03	0.34	0.29	0.2	0.27	0.48
COL4A3	−0.04	0.18	0.1	0.09	0.13	0.05	0.04	1	0.82	0.12	0.03	0	0.36	−0.04
COL4A4	0.05	0.24	0.22	0.23	0.25	0.02	0.03	0.82	1	0.08	0	−0.07	0.38	0.02
COL4A5	−0.25	−0.25	−0.23	−0.16	−0.17	0.26	0.34	0.12	0.08	1	0.76	0.17	−0.05	0.02
COL4A6	−0.38	−0.3	−0.11	−0.23	−0.1	0.15	0.29	0.03	0	0.76	1	0.22	0.04	−0.14
CA9	−0.34	−0.11	0.22	−0.11	0.06	0.18	0.2	0	−0.07	0.17	0.22	1	−0.09	−0.09
EPAS1	0.25	0.42	0.28	0.33	0.35	0.28	0.27	0.36	0.38	−0.05	0.04	−0.09	1	0.35
HSPG2	0.41	0.37	−0.17	0.17	0.07	0.44	0.48	−0.04	0.02	0.02	−0.14	−0.09	0.35	1
